# The Art Gallery Test: A Preliminary Comparison between Traditional Neuropsychological and Ecological VR-Based Tests

**DOI:** 10.3389/fpsyg.2017.01911

**Published:** 2017-11-17

**Authors:** Pedro Gamito, Jorge Oliveira, Daniyal Alghazzawi, Habib Fardoun, Pedro Rosa, Tatiana Sousa, Ines Maia, Diogo Morais, Paulo Lopes, Rodrigo Brito

**Affiliations:** ^1^Escola de Psicologia e Ciências da Vida, Lusophone University of Humanities and Technologies, Lisbon, Portugal; ^2^COPELABS–Cognition and People-Centric Computing Laboratories, Lisbon, Portugal; ^3^Faculty of Computing and Information Technology, King Abdulaziz University, Jeddah, Saudi Arabia; ^4^Department of Social and Organizational Psychology, ISCTE – University Institute of Lisbon, Lisbon, Portugal

**Keywords:** attention, serious games, virtual reality, ecological validity, cognitive assessment

## Abstract

Ecological validity should be the cornerstone of any assessment of cognitive functioning. For this purpose, we have developed a preliminary study to test the Art Gallery Test (AGT) as an alternative to traditional neuropsychological testing. The AGT involves three visual search subtests displayed in a virtual reality (VR) art gallery, designed to assess visual attention within an ecologically valid setting. To evaluate the relation between AGT and standard neuropsychological assessment scales, data were collected on a normative sample of healthy adults (*n* = 30). The measures consisted of concurrent paper-and-pencil neuropsychological measures [Montreal Cognitive Assessment (MoCA), Frontal Assessment Battery (FAB), and Color Trails Test (CTT)] along with the outcomes from the three subtests of the AGT. The results showed significant correlations between the AGT subtests describing different visual search exercises strategies with global and specific cognitive measures. Comparative visual search was associated with attention and cognitive flexibility (CTT); whereas visual searches involving pictograms correlated with global cognitive function (MoCA).

## Introduction

The use of virtual reality (VR) worlds within the field of mental health is a well-established reality. Since the 1990s, VR apps have sprouted up in every area of mental health; today, they cover the most common disorders and disabilities that are identified in the DSM 5 (APA, 2014): Posttraumatic Stress Disorder (PTSD), Obsessive-Compulsive Disorder (OCD), specific phobias and agoraphobias, depression, autism, Alzheimer’s disease, and acquired brain disorders. These conditions have all been trialed using VR worlds designed to better immerse patients in the therapeutic process (Brahnam and Jain, 2011).

Virtual reality worlds can reproduce any real-world scenarios – from shopping to taking a ride on the subway. In these worlds, participants can interact freely with the surrounding environment and characters, in the same manner as they do within a real-life environment (Cipresso et al., 2014). By standing in for reality, virtual environments provide ecological validity within a controlled environment, a combination that is missing from most of the traditional options available to treat mood disorders or to provide exercises for cognitive stimulation and assessment. For a review of the pros and cons of the use of VR applications within the field of mental health and rehabilitation (see Larson et al., 2014).

Another aspect that is also taken into consideration when choosing to use VR apps in treatments is their ability to assess what patients are doing in the virtual world. The system can be programmed to record everything that happens: trajectories, completion times, errors, and indecisions, among other indicators. The VR environment can incorporate in a natural way the actual obstacles that individuals with impairments must resolve in their daily routine. Improvements on indicators (e.g., less errors, lower completion times, less indecisions, and shorter trajectories) may indicate that the cognitive functions required to perform the required tasks were improved. This means that VR apps can assess and provide some insight on behavior performance of the user.

Although it is not possible to ensure their direct translation to a real-world situation, improvements in VR tasks that mimic real life are probably more reliable indicators of actual improvements in performance than are improvements on paper-and-pencil test scores (Silverberg and Millis, 2009; Parsons et al., 2013; Tarnanas et al., 2013; Oliveira et al., 2014; Aubin et al., 2015).

Nevertheless, the degree of functionality of a patient is usually evaluated with self-reports about everyday functioning in basic activities of daily living (ADL: measuring self-care skills), or instrumental activities of daily living (IADL: measuring independent living skills) (Graf and Hartford Institute for Geriatric Nursing, 2008).

Hence, paper-and-pencil tests that are developed under a construct-driven approach, which make them effective in assessing the cognitive constructs of interest, may be ineffective in predicting functional behavior as expressed in everyday tasks because they were not designed to assess actual performance of that behavior (Parsons et al., 2015).

One example of this is the assessment of attention during processes of selecting relevant information from the environment, which have been experimentally assessed through visual search tasks. One of the most used paradigms for this is the comparative visual search paradigm (Pomplun et al., 2001), which consists of a comparison between different shapes across two halves of a display. According to Galpin and Underwood (2005), such tasks involve visual attention for data acquisition, as well as memory to perform the comparison between the two images. Despite the importance of attention and memory for visual search, these tasks comprise the ability to plan and execute an organized pattern of behavior, which also depends on executive functions (Woods et al., 2013; Hardiess and Mallot, 2015).

Our study was built on these notions, as well as on those suggesting that neuropsychological instruments should be adapted to the demands of everyday life activities. We sought to develop a functional visual search task that consisted of an Art Gallery Test (AGT) within a virtual reality setting. This sort of VR-based assessment offers a contextually “realistic” environment that allows to study “real” psychological and behavioral responses (Bohil et al., 2011).

The AGT was devised to assess the cognitive processes involved in a comparative visual search task while performing matching exercises on paintings that are displayed side-by-side in an art gallery (Rosa et al., 2015, 2016). The three subtests of the AGT represented differences in task difficulty according to the strategy required from participants for visual search (as detailed in the Materials and Methods section).

The aim of the current paper was to study the relation between AGT and standard neuropsychological testing in a sample of adults, testing the hypothesis that performance on the AGT tests is related to the cognitive domains traditionally assessed by visual attention and cognitive functioning assessment scales, and therefore, be a more ecologically valid option compared to traditional neuropsychological testing. For that, the relationships between the AGT subtests with cognitive screening and attention tests were explored. The existence of those correlations can provide evidence to support the proposal of AGT as an alternative to traditional neuropsychological assessment.

## Materials and Methods

### Participants

The sample consisted of 30 university students (25 female) with a mean age of 25 years (*SD* = 8.20), all native Portuguese speakers recruited from a university campus in Lisbon, Portugal. Mean schooling of this sample was 14 years (*SD* = 1.9). Inclusion criteria were: (i) Portuguese native speakers, (ii) university students, and (iii) basic computer skills (mouse). The exclusion criteria were: (i) not having normal or corrected-to-normal vision, and (ii) current or history of psychiatric/neurologic disorders or substance abuse. No participants were excluded due to the exclusion criteria. Moreover, no significant differences were found between genders on age and education (*p* > 0.05).

### Measures

The measures used in this study consisted of paper-and-pencil neuropsychological tests and the AGT. The paper-and-pencil tests chosen were two screening tests for general cognitive ability and executive functioning and one specific test to assess attention and executive functions. These paper-and-pencil tests were used as concurrent measures of performance by the AGT. The screening tests are general tests that cover the most important domains of cognitive functioning, whereas the attention test assesses mostly divided and sustained visual attention, as also tested but in a different fashion in the AGT. The first screening test was the Montreal Cognitive Assessment (MoCA) developed by Nasreddine et al. (2005), which is one of the most used screening tests for cognitive impairments. The MoCA has been studied amongst distinct populations from different countries. It has also been validated for the general Portuguese population by Freitas et al. (2011). This test involves the most relevant cognitive domains that contribute to overall cognitive functioning; namely, executive functions, visuospatial abilities, memory, attention, concentration and working memory, language and orientation. Higher scores in this test reflect better cognitive function (ranges between 0 and 30).

Another goal was to explore the associations of the AGT with executive functions. Thus, the Frontal Assessment Battery (FAB) was also used in the current study. The FAB was developed by Dubois et al. (2000), and has been used ever since as a screening measure of executive functioning. This test assesses six different executive functions: (i) conceptualization, (ii) mental flexibility, (iii) motor programming, (iv) sensitivity to interference, (v) inhibitory control, and (vi) environmental autonomy. Higher scores on the FAB reflect better levels of executive functioning (ranged between 0 and 18).

The Color Trails Test (CTT) is a specific test for divided and sustained visual attention and executive functioning. This test was developed by D’Elia et al. (1996). The CTT consists of an A4 sheet with a number printed in two different colors. The participants in this test are instructed to link the numbered circles in the correct order with a pencil (i.e., increasing numeric order, alternating between colors) as fast as possible and without lifting the pencil from the paper. Two different forms of the CTT were used, which differ in their difficulty. The first trial of the test involves only numbers, whereas in the second trial the participants must shift between numbers and letters. We considered the number of errors (irrespective of the type) and completion time (in seconds). The interference index was also calculated to distinguish between tracking ability in CTT1, which assesses divided attention, and in CTT2 shifting between numbers and letters assesses executive function through cognitive flexibility (Dugbartey et al., 2000). The interference index was calculated according to the following expression:

(CTT2 completion time – CTT1 completion time)/CTT1 completion time)

Higher scores on both these measures are associated with poorer task performance. An additional executive score was also computed from a weighted composite score of the total score of the FAB, errors, and completion times of the CTT2 (weight FAB = 0.5 FAB; weight CTT2 errors = 0.25; weight CTT2 time = 0.25). Thus, the neuropsychological outcomes were global cognition (MoCA total score), executive score (FAB total score and CTT2 completion time), divided attention (CTT1 completion time), and interference index (CTT1 and CTT2 completion times).

**FIGURE 1:**
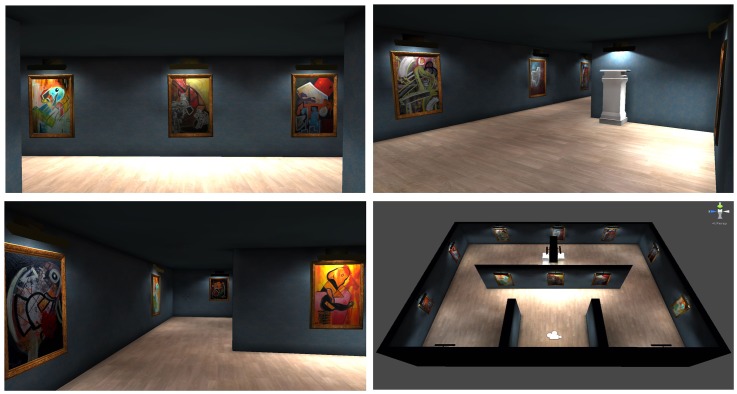
Screenshots from the Art Gallery Test (AGT).

**FIGURE 2:**
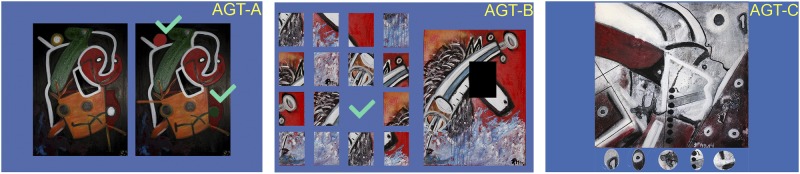
Examples of the AGT’s tasks: subtest A (AGT-A); B (AGT-B) and C (AGT-C).

The VR test used here was the AGT, developed with Unity 3D 4.6.4. The AGT was originally developed for the Systemic Lisbon Battery 2.0, which comprises several different tasks for cognitive assessment. The AGT consists of three sets of paintings (see **Figures 1**, **2**). In set 1 (subtest A), the participant must spot differences between two paintings that are displayed side-by-side; this was based on the comparative visual search paradigm (Pomplun et al., 2001), requiring the observer to compare the differences between the two halves of the display. Participants were instructed to find seven differences by clicking on the left mouse button. The second and third sets (respectively, subtests B and C) consist of simple visual search tasks with the target stimulus visible during task execution. In set 2 (subtest B), the participant is required to deconstruct a puzzle according to the details that are displayed on the left side, whereas in set 3 (subtest C), the participant is required to find five details in each painting, that are displayed below the painting; our intention was to create a specific attentional set in their visual search strategy. The design of subtests B and C of the AGT followed the methodology of Baddeley et al. (2001) visual search task using pictograms, but here using pieces of the paintings. Both of these subtests are based on visual search tasks, but subtest B also incorporates planning strategies.

Three different experts with expertise in neuropsychological assessment conceptualized the tasks that comprise the AGT. At this stage, the main goal was to develop a measure that gathers the main aspects of attention ability but with the functionalities of different tasks. The participants took a predefined path while moving from painting to painting (clockwise). The outcomes of this task were based on three different measures: (1) number of mouse clicks during the task (MC), (2) completion time in seconds (CT); and (3) composite scores, which represent the overall performance, by aggregating CT and MC scores that were calculated separately for subtest A, for subtest B, and for subtest C. CTs represent the total duration required to complete each task, since the exercise is displayed and until the participants ends it. It is, therefore, a measure of performance. MC number is an indicator of the number of tries a participant must make to successfully complete the task. A greater number of MCs might indicate that the participant is randomly clicking on the scenario, which represents a guessing-like behavior. In any of the subtests included in the AGT, the number of mouse clicks considered are the ones concerning the task itself, since the VR platform does not register any other clicks besides the ones regarding the task. CSs were the mean scores of MCs and CTs. However, they were applied independently to emulate the original paper-and-pencil tasks. Higher CS scores are supposedly associated with poorer performance.

### Procedure

The participants gave their written informed consent to the neuropsychological assessment conducted in this study. The participants were first assessed with the paper-and-pencil tests for neuropsychological assessment. A graduate student supervised by a senior neuropsychologist then assessed the participants with the neuropsychological tests. This assessment was conducted in a soundproofed and dimmed room in the experimental laboratory of the university from which the participants were recruited. After this assessment, the participants headed to another room where they were seated approximately 30 cm from a 17″ TFT monitor of an ASUS i7 CORE laptop computer with 2 GB GEFORCE GT Nvidia graphic board set up with the AGT. The paintings were displayed at the participants’ eye level. In the AGT, participants were instructed to look for the paintings and to perform the tasks as quickly as possible. The average time to complete each task was around 10 min. The interaction with the paintings, i.e., spotting the differences (subtest A), deconstructing the puzzle (subtest B), and finding the details (subtest C), was executed through clicking on the left mouse button. The left mouse was also used to move forward, and the right mouse button to move backward. The mouse movement emulated head movements within the virtual environment. Completion times and number of mouse clicks were generated to xls files. The total process (subtests A, B, and C) was concluded in approximately 30 min, after which the participants were dismissed.

Within subtest A, each pair of paintings displayed the same stimuli characteristics, such as luminosity, deepness and color. Within subtests B and C, the pieces/figures of the paintings that were used to deconstruct the puzzle (subtest B) and to be spotted on the paintings (subtest C) were retrieved from the painting that was under search, keeping, in this way, the original properties.

The paintings used are original work of a young Portuguese painter named João Marques (who is also a student at the School of Psychology and Life Sciences of ULHT) who agreed to have his work exhibited in our virtual gallery. Importantly, this ensures that the participants had no prior contact with the art pieces. All the work developed at LabPsiCom is freely available to other researchers on request. AGT has also been tested in touch-screen equipment, such as tablets and smartphones, and is also available on request.

### Statistical Analysis

The statistical analysis was performed with SPSS v.21. The first objective was to describe the central tendency, dispersion, and distribution of the neuropsychological and AGT indicators (CT, number of MC and CS, along with the *Z*-scores for each of the AGT outcomes). Following this analysis, we computed a composite factor, reflecting the mean number of mouse clicks and completion times, as previously explained. Correlations between these indicators and paper-and-pencil tests results were also calculated, as they can be forthcoming indicators of concurrent validity. This analysis was done for the global cognition (MoCA total score), executive score (FAB total score and CTT2 completion time), divided attention (CTT1 completion time), and interference index (CTT1 and CTT2 completion times). The significant correlations were then explored with a two-step hierarchical linear regression analysis. Given the small sample size, beta effects were estimated using bootstrap sampling with 95% confidence (5,000 samples) to compute robust coefficients (Efron and Tibshirani, 1993), and to investigate the predictive power of the most relevant predictors from the AGT while accounting for age effects.

## Results

### Descriptive Analysis on Neuropsychological Results

The descriptive analyses of the variables related to the neuropsychological assessment are shown in **Table 1**. The descriptive statistics were based on the mean scores and dispersion of the data through the standard deviation along with distribution statistics; namely, skewness and kurtosis for the variables from the MoCA, FAB, and CTT. MoCA total score is slightly lower than the normative data for the Portuguese population, according to the age and education levels of our sample (Freitas et al., 2011). Some of the distributions of the neuropsychological variables are negative in asymmetry, which may suggest ceiling effects, particularly in the naming and orientation subtest of the MoCA and all the subtests of the FAB, except for the conceptualization and mental flexibility. The variables concerning the CTT were adjusted to normal distribution apart from the interference index, which was positive in asymmetry with pronounced positive kurtosis.

**Table 1:** Descriptive analyses of the neuropsychological outcomes.

	*M*	*SD*±	Skewness	Kurtosis
**Montreal Cognitive Assessment**				
Visuospatial/executive	3.93	1.02	-0.71	0.50
Naming	2.97	0.18	-5.48	30.00
Attention	4.93	1.34	-0.89	-0.48
Language	2.43	0.68	-0.81	-0.40
Abstraction	1.83	0.38	-1.88	1.66
Delayed recall	2.53	1.53	0.06	-0.84
Orientation	5.93	0.25	-3.66	12.21
Total score	24.37	3.03	-0.19	-0.43
**Frontal Assessment Battery**				
Conceptualization	2.70	0.47	-0.92	-1.24
Mental flexibility	2.47	0.57	-0.46	-0.75
Motor programming	2.50	0.82	-2.01	4.16
Sensitivity to interference	2.87	0.43	-3.50	12.51
Inhibitory control	2.93	0.25	-3.66	12.21
Environmental autonomy	2.90	0.55	-5.48	30.00
Total score	16.37	1.75	-2.71	10.34
**Color Trails Test**				
CTT1 errors	0.00	0.00	-	-
CTT1 completion time	47.23	15.17	0.38	1.63
CTT2 errors	0.70	1.18	2.12	5.14
CTT2 completion time	97.70	29.52	0.85	-0.21
Interference Index	1.35	1.65	4.43	21.88


*CTT, Color Trails Test.*

### Descriptive Analysis on the AGT Outcomes

**FIGURE 3:**
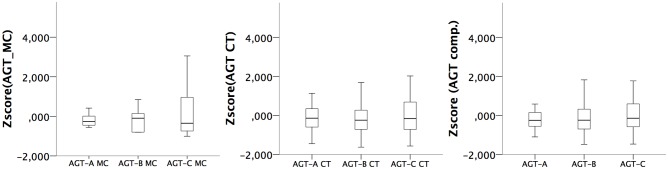
Boxplot charts for AGT-related variables.

The software generated two different measures (i.e., number of mouse clicks and completion times) for the three subtests. These two indicators were then transformed into composite scores for each subtest of the AGT. Thus, nine different variables were analyzed as outcomes of the AGT: three variables were related to the number of mouse clicks for each subtest; three variables for completion time, and three variables describing the composite scores for subtests A, B, and C. The distribution of these variables is shown through boxplot charts (**Figure 3**).

### Bivariate Pearson Correlations of AGT’s Indicators with Neuropsychological Outcomes

The AGT’s correlations with standard neuropsychological outcomes were computed with the Pearson *r* coefficient (**Table 2**). The neuropsychological outcomes consisted of global cognition (MoCA total score), executive score (FAB total score, CTT2 errors and completion time), divided attention (CTT1 completion time), and interference index (completion times for CTT1 and CTT2). This analysis showed a correlation between the AGT-A with the interference index from the CTT (*r* = 0.452; *p* = 0.012), and between the AGT-C with global cognition as assessed with the MoCA (*r* = -0.420; *p* = 0.021), suggesting that interference (less cognitive flexibility) is associated with a poorer performance in the AGT-A (i.e., higher scores: more time, more mouse clicks), whereas a poorer performance in the AGT-C is associated with poorer global cognitive function (lower MoCA scores).

**Table 2:** Bivariate Pearson (*r*) correlation coefficients between the AGT and neuropsychological outcomes.

	AGT-A total	AGT-B total	AGT-C total
Global cognition	-0.072	-0.138	-0.420^∗^
Executive score	0.220	0.070	0.005
Divided attention	-0.038	0.041	0.013
Interference index	0.452^∗^	0.001	-0.015


*^∗^*p* < 0.05. The variables in the first row are related to composite scores for each subtest of the AGT. The variables in the left column are the global cognition retrieved from the Montreal Cognitive Assessment, the executive score calculated from the Frontal Assessment Battery and the Color Trails Test – trial 2, divided attention from the Color Trails Test – trial 1, and the interference index from the Color Trails Test.*

### Prediction of Cognitive Performance

As considerable evidence has shown that lifespan-associated developmental change is an important covariate of cognitive performance (Rosa et al., 2017), age was included in the first step of the hierarchical regression. In the second step, the composite score of AGT in subtest C was added in the statistical model, since this was the only potential predictor found. The **Table 3** displays the results of the regression analysis. In the first step, age was not a significant predictor of MoCA *B* = -0.05; 95% CI [-0.36, 0.07]; *SE* = 0.13, explaining only 2% of the variance on MoCA. However, when AGT-C total was introduced in the second step, the regression model became significant, accounting for 18% of the total variance in MOCA. The AGT-C total was a significant predictor of MoCA *B* = -0.45; 95% CI [-0.77, 0.01]; *SE* = 0.20, after controlling for age.

**Table 3:** Summary of the Bootstrapped Hierarchical Regression Analysis when predicting global cognition and interference index.

Predictor	Criterion variable	*R*^2^	*F*	Δ*R*^2^	Δ*F*	*B*	*SE*	CI 95%
*Step 1*	Global cognition	0.02	0.46 (1, 28)					
Age						-0.05	0.13	[-0.36, 0.07]
*Step 2*		0.18	2.93 (2, 27)	0.16	5.33^∗^ (2, 27)			
Age						-0.02	0.12	[-0.34, 0.09]
AGT-A						-0.44^∗^	0.20	[-0.77, -0.01]
*Step 1*	Interference Index	0.03	0.82 (1, 28)					
Age						0.03	0.08	[-0.04, 0.27]
*Step 2*		0.23	3.97 (2, 27)	0.20	6.95^∗^ (2, 27)			
Age						-0.04	0.07	[-0.18, 0.13]
AGT-C						-0.03	0.03	[-0.01, 0.08]


*^∗^*p* < 0.05. *N* = 30 Values inside the parenthesis are the degrees of freedom. The Δ*R*^*2*^ and *F*-values were derived from hierarchical regression analysis. Bootstrap results are based on 5,000 resamples.*

The same analysis was conducted for the AGT-A predicting the interference index, but no significant effects were found in this model. Age was not a significant predictor of the interference index in the first step *B* = 0.03; 95% CI [-0.04, 0.27]; *SE* = 0.08. The second step was significant with the inclusion of AGT-A in the model (21% total variance), although the Beta score for AGT-A remained non-significant, *B* = -0.03; 95% CI [-0.01, 0.08]; *SE* = 0.03.

## Discussion

The main goal of this study was to conduct a pilot study to investigate the opportunity to use an ecological neuropsychological test – AGT, as an alternative to traditional paper-and-pencil tests.

Traditional cognitive assessment and rehabilitation materials lack ecological validity and are far from pleasant for patients who have impaired cognitive functions, and need to repeat the same exercise on a daily basis to recover cognitive ability or to minimize the impact of compromised functions (Larson et al., 2014). VR can produce meaningful, ecologically valid, and motivating environments, in which patients can exercise in a gaming fashion (Graf and Hartford Institute for Geriatric Nursing, 2008; Gamito et al., 2011). The AGT follows these principles, and aims to be an alternative to complement the results from traditional assessment.

To meet this aim, execution scores from a non-clinical population are required. These values represent the average amount of completion time and the average number of attempts (number of mouse clicks) that a healthy participant would take to complete the task at hand. From these results, it would then be possible to find, in future studies with sub-clinical and clinical populations, probable deviations that are likely to occur when a non-healthy participant performs the same task.

The associations between the AGT subtests with the neuropsychological outcomes showed a relationship between subtest A and the interference index of the CTT. These results suggest that the comparative visual search task that was considered more difficult (higher proportion of number of mouse clicks and completion times) was more associated with language-free visual attention processes and cognitive flexibility, as measured in the CTT (Dugbartey et al., 2000), but probably due to the small sample size, the predictive ability of this subtest on the interference index was not significant.

The correlations showed also an association between subtest C, with global cognition as assessed with the MoCA, which was further confirmed using linear regression analysis. The result on subtest C may mean that performance on functional VR tasks is difficult to discriminate through paper-and-pencil tests, as this performance may involve global cognition rather than a specific cognitive function (Oliveira et al., 2017). This was indeed found for the execution of the subtest C of the AGT, which described a visual search task to find the details using pieces of paintings. However, no other associations were found between AGT and executive score, divided attention, nor yet subtest B, which is intriguing given the similarities between subtests B and C. Both tasks were developed using the visual search paradigm, but, considering the visual properties of each of these subtests, the execution strategy may have differed between them. The squared target items in subtest B may have been identified more easily than the small pieces of the paintings in subtest C. A visual inspection of **Figure 3** suggests that mouse clicks were lower on subtest B than on subtest C, which may be indicative of different execution strategies to accomplish these tasks. The AGT will require further investigation in clinical samples, with less ceiling effects on neuropsychological data, to better understand the differences between AGT subtests, and whether task difficulty is adjusted to patients with cognitive impairments.

One of the limitations of this study is the small sample. This study was intended as a preliminary approach to the study of AGT as a robust and ecologically valid alternative to traditional neuropsychological assessment. Therefore, the sample size was defined as the minimum necessary to comply with parametric testing based on the central limit theorem. However, the authors will continue this study after proceeding with some technical adjustments and general improvements based on the results of this preliminary study. Another issue concerns gender imbalance, which is justified by the fact that this is a convenience sample from Psychology school where most students are women. Nevertheless, gender was not a factor in the results since the authors previously controlled for its effect. Again, further research with AGT will also take this into consideration.

Although the three AGT subtests were developed to assess attention, the results from this study suggest that the demands of each task and the way they are executed influence the underlying cognitive ability required to accomplish the task (Parsons et al., 2015). Only subtest A correlated with interference index from an attention measure (albeit this effect was not supported in the regression). Subtest C worked as a global measure of cognition, not discriminating what specific cognitive abilities were involved in the execution of this task.

Overall results were more consistent in associating visual search using pictograms (subtest C) with global cognition, although VR comparative visual search (subtest A) may be associated with visual attention and cognitive flexibility as assessed in the CTT.

## Ethics Statement

The study protocol was approved by the EPCV Ethics Committee. All participants gave their written informed consent.

## Author Contributions

PG was responsible for the first draft of the manuscript, whereas the literature searches and summaries were performed by JO. PG, JO, DM, and PL were involved in the conception of the study, whereas PR, TS, IM, DA, and HF were involved in the study design. PG, DA, and HF were responsible for the technological input to this project. TS carried out neuropsychological evaluations and PR did the statistical analysis. PG, JO, PR and RB have contributed to data interpretation. DM, PL prepared the evaluation protocol. PG, JO, DA, HF, DM, and PL were also responsible for text revision, with PG being the responsible for the final version of the manuscript. All authors contributed to and have approved the final version of the manuscript.

## Conflict of Interest Statement

The authors declare that the research was conducted in the absence of any commercial or financial relationships that could be construed as a potential conflict of interest.
